# Classification of subtask types and skill levels in robot-assisted surgery using EEG, eye-tracking, and machine learning

**DOI:** 10.1007/s00464-024-11049-6

**Published:** 2024-07-22

**Authors:** Somayeh B. Shafiei, Saeed Shadpour, James L. Mohler, Eric C. Kauffman, Matthew Holden, Camille Gutierrez

**Affiliations:** 1 The Intelligent Cancer Care Laboratory, Department of Urology, Roswell Park Comprehensive Cancer Center, Buffalo, NY 14263 USA; 2 Department of Animal Biosciences, University of Guelph, Guelph, ON N1G 2W1 Canada; 3 Department of Urology, Roswell Park Comprehensive Cancer Center, Buffalo, NY 14263 USA; 4 School of Computer Science, Carleton University, 1125 Colonel By Drive, Ottawa, ON K1S 5B6 Canada; 5 Obstetrics and Gynecology Residency Program, Sisters of Charity Health System, Buffalo, NY 14214 USA

**Keywords:** Cystectomy, Hysterectomy, Nephrectomy, Dissection

## Abstract

**Background:**

Objective and standardized evaluation of surgical skills in robot-assisted surgery (RAS) holds critical importance for both surgical education and patient safety. This study introduces machine learning (ML) techniques using features derived from electroencephalogram (EEG) and eye-tracking data to identify surgical subtasks and classify skill levels.

**Method:**

The efficacy of this approach was assessed using a comprehensive dataset encompassing nine distinct classes, each representing a unique combination of three surgical subtasks executed by surgeons while performing operations on pigs. Four ML models, logistic regression, random forest, gradient boosting, and extreme gradient boosting (XGB) were used for multi-class classification. To develop the models, 20% of data samples were randomly allocated to a test set, with the remaining 80% used for training and validation. Hyperparameters were optimized through grid search, using fivefold stratified cross-validation repeated five times. Model reliability was ensured by performing train-test split over 30 iterations, with average measurements reported.

**Results:**

The findings revealed that the proposed approach outperformed existing methods for classifying RAS subtasks and skills; the XGB and random forest models yielded high accuracy rates (88.49% and 88.56%, respectively) that were not significantly different (two-sample t-test; *P*-value = 0.9).

**Conclusion:**

These results underscore the potential of ML models to augment the objectivity and precision of RAS subtask and skill evaluation. Future research should consider exploring ways to optimize these models, particularly focusing on the classes identified as challenging in this study. Ultimately, this study marks a significant step towards a more refined, objective, and standardized approach to RAS training and competency assessment.

RAS has emerged as a promising field that combines the precision and dexterity of robotic systems with the expertise of surgeons. As this technology is deployed more widely, there is a growing need to develop intelligent systems that can accurately assess and classify both the subtasks performed by surgeons and their skill levels. Such capabilities would provide objective feedback to surgeons and contribute to enhancing training programs and improving patient outcomes [1].

Researchers have integrated multiple modalities, including electroencephalogram (EEG) and eye-tracking, and advanced machine learning (ML) algorithms [2–4] to develop RAS skill level evaluation models. EEG captures the brain activity to provide information about the cognitive and motor processes involved in performing a surgical task [5, 6]. Eye-tracking allows non-intrusive measurement of visual attention to enable assessment of a surgeon’s focus and attention during a procedure [7–9]. EEG offers the potential to explore a surgeon’s cognitive workload during RAS, which provides insight into their concentration, fatigue, and stress levels [4]. Studies have reported correlations between EEG patterns and cognitive workload in surgeons, with specific patterns linked to high cognitive workload [4]. Experienced surgeons typically demonstrate efficient and purposeful eye movements, while less experienced surgeons may exhibit more frequent and random eye saccades. Thus, analyzing eye-tracking patterns can offer information about a surgeon’s expertise level and cognitive strategies [3].

ML and Deep learning (DL) algorithms play a pivotal role in the classification of subtasks and skill levels in RAS. One potential advantage of employing ML and DL is their ability to help reduce individual human biases, particularly by providing objective, data-driven assessments that can standardize evaluations across different operators. ML encompasses a broad range of algorithms and methodologies that enable computers to learn from and make predictions based on data, including both simple algorithms like linear regression and more complex ones like decision trees. DL, a subset of ML, specifically refers to models that utilize deep neural networks, characterized by multiple layers that enable the learning of highly abstract features of data automatically. These networks often require larger datasets and more computational power than traditional ML approaches. Unlike many traditional ML methods, which may require manual feature identification and adjustment, DL models can automatically learn and improve from their own errors, making them highly effective for complex tasks. By leveraging the multimodal data collected from EEG and eye-tracking, these algorithms can identify patterns that discriminate between different subtasks and skill levels. Through a combination of supervised learning and feature selection techniques, the ML models can learn from labeled data and generate predictive models to classify surgical subtasks. These methods involve training a ML model on a labeled dataset, where the ‘labels’ represent the various surgical skill levels. Once trained, the model can then classify new, unseen data into these categories. This process has distinguished inexperienced, competent, and experienced RAS surgeon skill levels based on EEG and eye-tracking data [3, 10].

*Advantages of classifying both subtasks and skill levels in RAS* Classifying surgical subtask type and skill level together offers several potential advantages:

1. Comprehensive assessment: The classification system can provide a more comprehensive assessment of a surgeon’s performance during a procedure by considering both the specific actions performed (subtask type) and the proficiency with which they were executed (skill level).
2. Objective evaluation: Subtle differences in cognitive processes and motor planning associated with different subtask types and skill levels can be captured by analyzing neural activity. This feature minimizes subjective biases that may be present in traditional manual assessments.
3. Training and skill development: A model that classifies surgical skill levels using patterns of EEG and eye-tracking data could notably improve surgical training. If validated and generalized, the system could integrate into current programs, providing trainees with objective feedback on their performance in conducting surgical tasks. It would serve as an additional support to direct supervision and evaluation, thereby enhancing the efficiency of RAS training. This approach promotes focused practice, potentially accelerating the learning process and shortening the overall training duration.

This study explores the classification of RAS subtasks and skills using EEG, eye-tracking, and ML algorithms. The combination of these technologies offers a promising approach for creating a holistic evaluation of a surgeon’s expertise, potentially influencing surgical education, training, and operative performance [11, 12].

## Methods

This study was conducted in accordance with relevant guidelines and regulations and was approved by the Institutional Review Board (IRB: I-241913) and Institutional Animal Care and Use Committee approval (IACUC 1179S) of the Roswell Park Comprehensive Cancer Center. The IRB issued a waiver of documentation of written consent. All participants were given a research study information sheet and provided verbal consent.

*Participants and tasks* This study encompassed eleven physician participants, which included ten males and one female, with average age of 42 ± 12 years. The participants included two physicians training in a surgical residency, four surgeons training in a specialty fellowship, and five fellowship-trained surgeons specialized in gynecology, urology, or thoracic surgery.

**Fig. 1:**
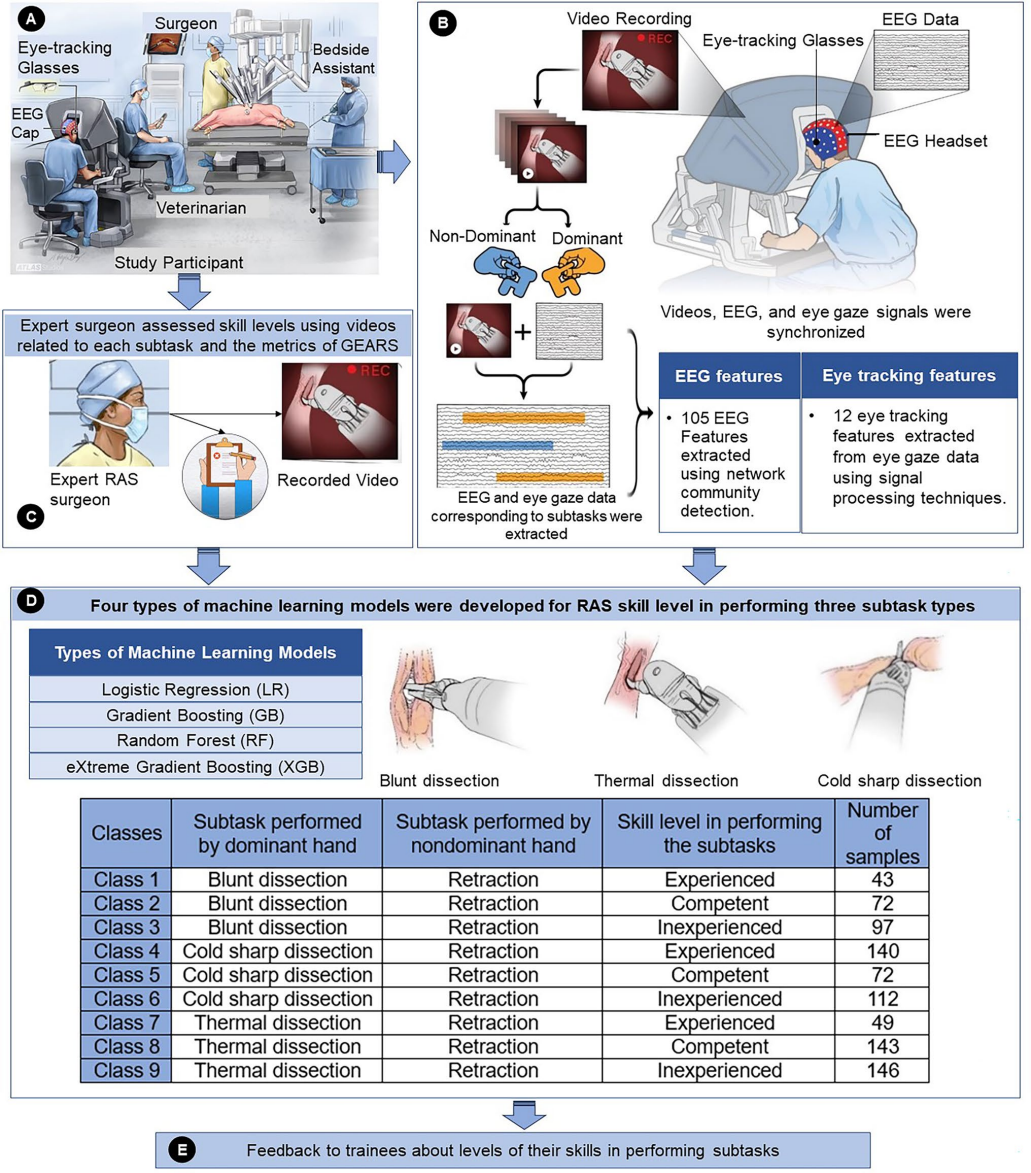
Representation of **A** operating room and data recording, **B** synchronizing EEG, eye-tracking, and operation videos, and extraction of data associated with surgical subtasks performed by dominant and non-dominant hands, **C** assigning actual skill level in performing subtasks by each participant, **D** model development using inputs to evaluate subtask type and skill level, **E** output

Participants performed 11 hysterectomies, 11 cystectomies, and 21 nephrectomies using the da Vinci surgical robot on live pigs (IACUC 1179S). Operations were performed during one session that lasted for four to six hours. An expert RAS surgeon attended the session as the mentor if a participant did not have operative experience. A veterinarian assisted in the set-up and provided oversight for the animal welfare (Fig. 1a).

For the hysterectomy operation, participants accessed the pelvic cavity, isolated the uterus, ligated blood vessels, detached the uterus, and removed it through a skin incision, prioritizing precision. The cystectomy operation involved, dissecting around the bladder and releasing bladder attachments, clamping and cutting the urethra, and extracting the bladder, maintaining visualization and instrument control. For the nephrectomy, access was gained to the retroperitoneal space to visualize the kidney, followed by its separation from adjacent organs, transection of the renal artery and vein, and removal of the kidney through a skin incision, requiring precise maneuvering of robotic instruments.

*Surgical subtasks* The operative videos were analyzed to determine the start and end times of three primary subtasks—blunt, cold sharp, and thermal dissection—performed by the dominant hand, alongside retraction by the non-dominant hand. In hysterectomies, blunt dissection separates the uterus from connective tissues and cold sharp dissection ligates blood vessels, with thermal dissection less common due to nearby delicate structures. For cystectomies, blunt dissection is used for initial exploration and isolating the bladder, while cold sharp dissection minimizes blood loss, and thermal dissection seals vessels cautiously due to nearby critical structures. In nephrectomies, blunt dissection isolates the kidney and identifies vessels, cold sharp dissection thoroughly dissects the renal hilum, and thermal dissection secures and splits vessels, ensuring minimal adjacent damage.

*Data recording* EEG data were recorded from participants using a 124-channel AntNeuro ® EEG system at 500 Hz. Eye-tracking data were recorded using Tobii ® eyeglasses at 50 Hz (Fig. 1).

**Fig. 2:**
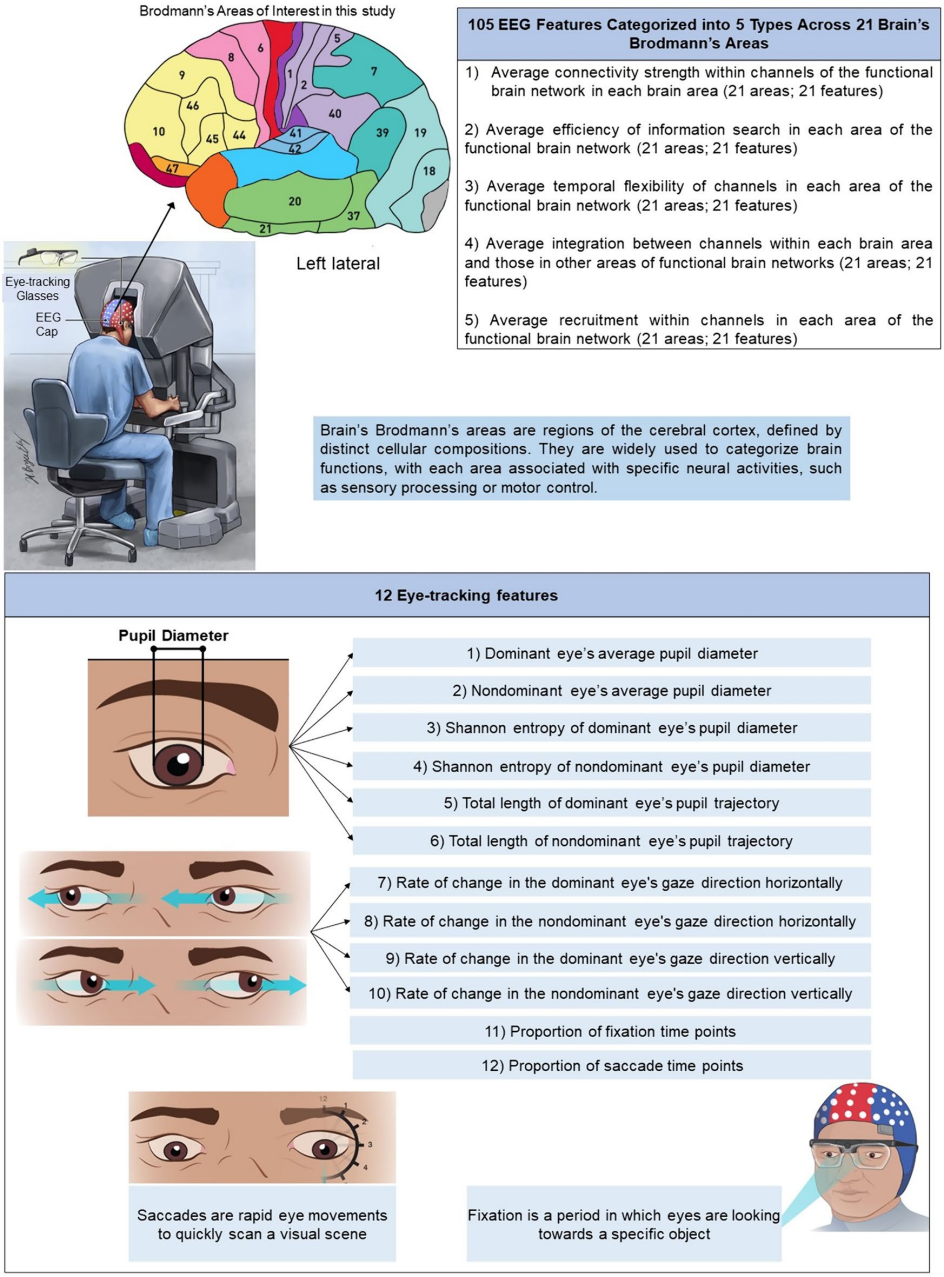
Representation of 105 features extracted from EEG data and 12 features extracted from eye-tracking data

*EEG features* EEG data were preprocessed and decontaminated from artifacts using the approach in our previous study [13–16]. Post-decontamination, coherence analysis was conducted to derive the functional brain network. Key EEG features (Fig. 2) [17] were extracted using established approaches from our prior research [13, 14]. These metrics assist in understanding the brain’s information processing mechanisms during surgery. For instance, search information provides information about the efficiency of information transfer across different brain regions, while strength demonstrates the effectiveness of communication among various brain areas [18–20]. Flexibility facilitates comprehending how the brain adapts over time in response to varying demands [21, 22]. In the surgical context, higher flexibility might correlate with the surgeon’s capacity to respond to unexpected intraoperative events. Integration explains how different brain areas collaborate over time [23]. Recruitment represents the activation of specific brain areas that form interconnected networks while performing cognitive or behavioral tasks [24, 25]. This pattern of brain network recruitment can provide crucial information about the neural mechanisms highlighting different cognitive functions and can assist to understand how the brain processes information and produces behavior. The selection of these features was strategic, aimed at enhancing the understanding of the brain’s information processing dynamics specifically during RAS.

*Eye-tracking features* Eye-tracking data were used to extract visual features using the approach in our previous study [3]. Those features are defined in Fig. 2.

*Actual skill levels* The modified Global Evaluative Assessment of Robotic Skills (GEARS) was used by an expert RAS surgeon (J.L.M.) to assess participants’ surgical skills through recorded operation videos. GEARS, designed for RAS technical skill evaluation, measures six dimensions: depth perception, bimanual dexterity, efficiency, force sensitivity, robot control, and autonomy, each rated on a 1 to 5 Likert scale, resulting in total scores ranging from 6 to 30 [26]. GEARS categorizes surgical expertise into three levels: inexperienced, competent, ad experienced. The expert rater assigned scores for each dimension and determined the skill level, which was then used to assign a specific actual skill level to each subtask. The synchronization of subtasks and skill levels is detailed in Fig. 1.

### ML models development

EEG and eye-tracking features for each surgical subtask were extracted, and, along with actual skill levels and subtask types, fed into ML algorithms including multinomial logistic regression (MLR) [27], gradient boosting (GB) [28], random forest (RF) [29], and extreme gradient boosting (XGB) [30]. The objective was to create models capable of classifying nine classes, representing a combination of subtask type and skill level. Details about each algorithm’s attributes and hyperparameter values are provided in Appendix 1.

*Training and testing* To validate our model, we adopted a strategy where 20% of the samples from each class were randomly selected and held out as a test set, while the remaining 80% of samples formed the training and validation sets. This approach was chosen due to the unique challenges involved in developing a RAS skill level classification model, particularly in clinical studies within operating room settings. Some of these challenges include: (1) a limited number of participants; (2) variation in the number of subtasks each participant performs to complete a surgical task; (3) fluctuating skill levels of participants from one operation to another, affecting their proficiency in specific subtasks. These factors complicated the use of more complex training techniques such as leave-one-subject-out cross-validation, primarily due to severe class imbalance. Consequently, a train-test split performed over 30 iterations was determined to be the most feasible approach for this dataset, balancing the need for robust data handling while mitigating potential biases associated with severe class imbalance. Models were then trained and validated using a grid search technique combined with stratified cross-validation (fivefold cross-validation repeated five times), effectively preventing model overfitting and accounting for variability in surgical performances.

The Synthetic Minority Over-sampling Technique was employed on *training set* to mitigate the issue of class imbalance in data sample categories [31]. This process was repeated 30 times. The average performance across these iterations was reported. For hyperparameter tuning, we employed a grid search technique combined with stratified cross-validation (fivefold cross-validation repeated five times). This involved exploring a range of values and selecting the best combination based on accuracy (Appendix 1).

### Evaluation of ML models

The performance of the models in classifying subtask and surgical skill levels was assessed using various statistical metrics. Precision: The proportion of accurate positive predictions to the sum of all predicted positive outcomes by the classifier; Recall (Sensitivity): the fraction of correct positive predictions to the sum of all actual positive results in the dataset; Accuracy: The ratio of accurate predictions to the total quantity of predictions made; F-Score: constitutes the harmonic mean of precision and recall, oscillating between 0 and 1, where a superior score signifies better performance. Confusion Matrix: Rows of this matrix correspond to the actual classes and its columns correspond to the predicted classes.

*Comparison of models’ performances* To determine whether the results of each model were significantly different from each other, a two-sample t-test was applied to the pairs of accuracy results derived from 30 iterations of each model. The Bonferroni *p*-value correction was applied for conducting six comparisons for pairs of four models.

## Results

**Fig. 3:**
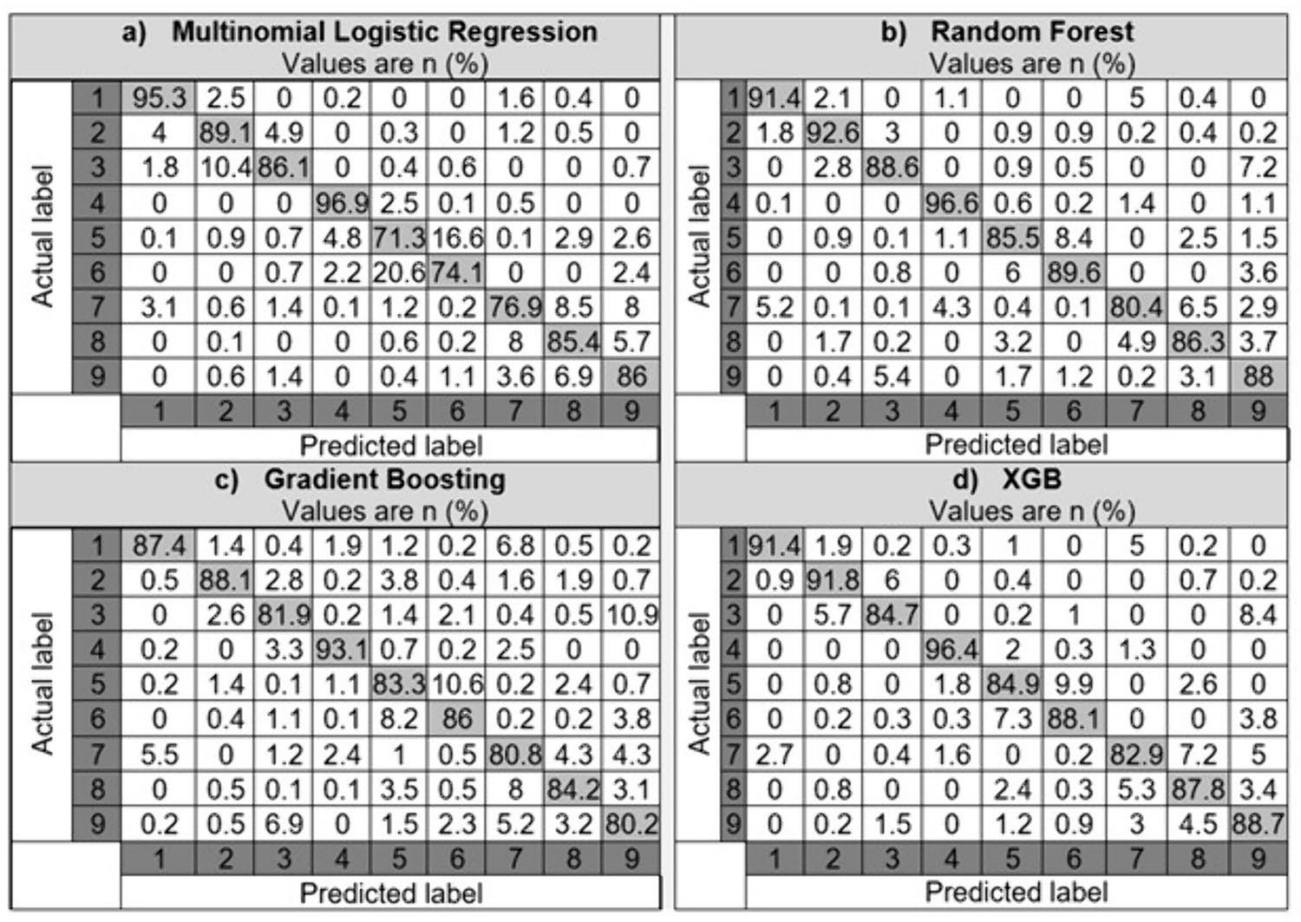
The confusion matrices for machine learning classification models employed in predicting subtask type and skill levels

In the category of blunt dissection subtasks, experienced participants executed 43, while those considered competent performed 72, and the inexperienced group completed 97. Regarding cold sharp dissections, experienced participants conducted 140, competent participants performed 72, and those inexperienced carried out 112. Finally, for thermal dissections, the experienced, competent, and inexperienced participants performed 49, 143, and 146, respectively. A random selection of 20% of the samples from each class was reserved as a test set. The actual class of these test samples, encompassing both skill level (assessed by an expert RAS surgeon) and subtask type (from operation videos), was then compared with the classifications made by the developed models. The outcomes of this comparison, including various statistical metrics and confusion matrices, expressed as percentages (%), are presented in Table 1 and Fig. 3, respectively. Table 1 presents the efficacy of various ML classification models (MLR, RF, GB, and XGB) in predicting subtask type and skill level, using key performance metrics like Precision, Recall, Accuracy, Specificity, and F1 Score.

**Table 1:** Efficacy of ML classification models in predicting subtask type and skill level

	Multinomial logistic regression	Random forest	Gradient boosting	XGB
Precision (%)	84.16	88.65	85.23	88.81
Recall (%)	84.57	88.78	85	88.52
Accuracy (%)	83.81	88.56	84.95	88.49
Specificity (%)	97.96	98.56	98.11	98.55
F1score (%)	84.3	88.68	85.1	88.63

The RF and XGB models showed superior performance across all metrics, indicating their robustness and effectiveness in classifying subtask types and skill levels. The reasonably high scores in specificity across all models are particularly notable, underscoring their reliability in a medical context.

Each model generally performs well in classifying the subtask types and skill levels, with the diagonal cells (indicating correct classifications) showing high percentages. For instance, in most models and classes, the accuracy ranges from the high 70 s to mid-90 s in percentage terms. The off-diagonal cells in Fig. 3 indicate instances where the model misclassified a subtask or skill level. These are relatively low for all models, suggesting that the models are quite robust.

*Analyzing the precision of classification models* The results of two-sample t-tests showed that RF is significantly better than MLR (*p* < 0.001) and GB (*p* < 0.001), but its performance is not significantly different from XGB’s performance (*p* = 0.9). The XGB model performed significantly better than both LR and GB (both *p* < 0.001). However, after Bonferroni correction for multiple comparisons, the difference in performance between GB and LR was not statistically significant (*p* = 0.02).

## Discussion

Research regarding surgical skill assessment is mainly focused on evaluating skill across entire surgical procedures, predominantly utilizing kinematic data and video recordings (Table 2) [32–38]. EEG’s high temporal resolution captures the dynamic cognitive processes underlying surgical tasks, offering insights beyond the external movements analyzed in video data. By measuring core neural mechanisms like attention, cognitive load, and decision-making, EEG provides a deeper understanding of surgical skills. By integrating EEG with eye-tracking technology, this approach offers a more comprehensive perspective that reveals aspects of surgical skill that are not detectable through current machine learning and deep learning models developed using only video data. These models primarily analyze video data, focusing mainly on the kinematics of the surgeon’s hand movements.

**Table 2:** State-of-the-art studies proposing surgical skill and subtask classification models

Author	Year	Population	Setting	Tasks	Data	Classes	Model^*^	Accuracy
Wang Y. et al. [35]	2021	18	RAS, laboratory setting	suturing	video recordings	skill level: novice, intermediate, expert	DL	83%
Soangra et al. [36]	2022	26	laparoscopic simulator and RAS, laboratory setting	peg transfer, knot tying	kinematic data and electromyogram	skill level: novice, intermediate, expert	ML	58%
Law et al. [37]	2017	29	RAS, operating room	robotic prostatectomy	video recordings	skill level: binary (good vs. poor)	DL, ML	0.92
Natheir et al. [38]	2023	21	three simulated brain tumor resection procedures on the neuroVR™ platform, laboratory setting	brain tumor resection procedures	EEG	skill level: binary (skilled vs. less skilled)	ML	85%
Zappella et al. [39]	2013	8	RAS, laboratory setting	suturing, needle passing, knot tying	video and kinematic data	task detection: suturing, needle passing, knot tying	DL, ML	80%–94%
Wang et al. [40]	2018	8	RAS, laboratory setting	suturing, needle passing, knot tying	video and kinematic data	skill level: novice, intermediate, expert	DL	91%–95%
Current study	2024	11	RAS, operating room	blunt, cold sharp, and thermal dissection subtasks throughout cystectomy, hysterectomy, and nephrectomy operations	EEG and eye-tracking	skill level (inexperienced, competent, experienced) and subtask type (blunt, cold sharp, and thermal dissection); 9 classes	ML	83%–88%

*ML Machine Learning, *DL* Deep Learning

Research in the domain of surgical skill and subtask classification predominantly bases on simulated tasks or exercises undertaken on plastic models in laboratory environments. Comparative studies conducted in clinical settings are often constrained by a predominant emphasis on assessing outcome metrics, as opposed to a detailed analysis of individual subtasks [41, 42]. Understanding surgical skill requires a granular analysis of individual surgical subtasks, essential for standardizing expertise assessment and interpreting the interrelations among various tasks. This approach not only enhances our understanding of surgical performance but also holds potential in improving patient outcomes [43].

Findings of this study demonstrated that ML classifiers trained by EEG and eye-tracking features can predict the type of surgical subtask and skill level based on EEG and eye-tracking features with a reasonable accuracy. The models showed promising results in classifying different surgical subtask types and skill levels, with certain areas that could benefit from further model optimization or feature refinement. The consistency in high performance across different models also reinforces the robustness of the underlying features used for model training. The misclassifications occurred in some classes (e.g., Class 5 and Class 6) could be due to the inherent difficulty in distinguishing these classes or due to overlapping characteristics between them. The variability in misclassification patterns across models suggests that integrating these models or stacking them could potentially improve the overall predictive performance.

Findings of this study are in agreement with the conclusions drawn by a number of previous studies, which posit that XGB and RF models often outperform other algorithms, primarily due to their robustness and ability to handle diverse datasets [29, 44].

Despite the inherent challenges in facilitating a thorough and equitable comparison with contemporary state-of-the-art studies—attributable to differences in task specifications, actual skill level assignment, methodologies, and ML training/validation strategies—the present study surpassed some of the previously documented highest accuracy rates in RAS skill classification, particularly in the context of operating room procedures [32, 33]. Chen et al. analyzed kinematic data from 17 participants executing 68 vesico-urethral anastomosis procedures. They trained AdaBoost, GB, and RF algorithms to differentiate between two skill levels: expert and novice. Utilizing 80% of their data for training and the remaining 20% for testing, they compared the actual skill levels of the test samples against the predictions of their models, achieving 77.40% accuracy (i.e., their model detected skill level of 77.40% of test samples correctly) [32].

### Clinical applications of findings

This area of research is still emerging, but the classification of subtask type and skill levels in RAS utilizing EEG, eye-tracking, and ML holds significant promise for the future of surgical education and training. More detailed and quantitative measurement of RAS skills acquisition may provide an opportunity for objective feedback regarding skill level, which would enhance the RAS training and improve patient safety. This study introduced ML models that can do this by providing opportunities for better skill assessment and training programs, possibly leading to the creation of personalized training plans.

Broader implications of the validated ML models for assessing surgical skills: These models provide a reasonably accurate method to assess the expertise levels of surgeons across a spectrum from inexperienced to experienced, and hold potential to shape milestones in surgical education. For example, they can serve as reliable metrics for determining graduation readiness in residency and fellowship programs. Furthermore, they provide a robust baseline for credentialing, ensuring that surgeons meet standardized competence levels before they practice independently. Such applications could markedly enhance the quality of surgical training and patient care, positioning the developed models as important additions to surgical education and professional development frameworks.

*Strengths* This study’s key strength lies in combining EEG and eye-tracking data from surgeons and trainees to build models that can evaluate surgical skills and concurrently identifying the ongoing subtask. The study integrates data from both EEG and eye-tracking features, offering a comprehensive view of the surgeon’s performance from multiple perspectives, which could lead to a more accurate classification of skill levels and subtask types. The ML model facilitates an objective evaluation of surgical skills, potentially reducing subjectivity and bias in skill assessments, which is a significant step forward in surgical training and performance evaluation. The study lays the groundwork for the development of personalized training modules, allowing for targeted improvement of specific skills based on objective assessments of individual strengths and weaknesses.

*Limitations* While the results are promising, further studies should aim for a more varied range of subtasks and a larger, more diverse participant pool to enhance generalizability. Also, the participant pool was heavily skewed towards male participants. Future research should focus on including participants of varying expertise and gender to aid in developing more universally applicable models. Additionally, the slight differences in animal and human surgical anatomy could affect the outcomes, suggesting a need for patient-based validation to confirm these findings. In line with previous studies, which established that the integration of raw data alongside engineered features in a deep neural network model can enhance skill assessment precision [45], future exploration will investigate the potential of augmenting results through the application of raw data in a deep neural network model.

## Data Availability

Data supporting the findings of this study are available from the corresponding author (S.B.S.) upon reasonable request.

## Appendix 1

*Multinomial Logistic Regression (MLR)* Logistic regression is a statistical method for modeling the relationship between a categorical dependent variable and one or more independent variables. When the dependent variable is nominal with more than two classes, the variant of logistic regression used is called MLR [27]. Nominal variables are categorical variables with two or more categories without any intrinsic ordering. MLR is a linear classification method that models the probability of an instance belonging to a specific class through a logistic function, which is an S-shaped curve that can take any real-valued number and map it between 0 and 1. This makes it a suitable representation for probability. Key hyperparameters for MLR tuning include (1) Penalty: Represents the type of regularization applied to prevent overfitting. The possible values are ‘l1’, ‘l2’, or ‘elasticnet’. Regularization adds a penalty on the different parameters of the model to reduce the freedom of the model and prevent overfitting; (2) C: Denotes the inverse of regularization strength. A smaller value of C means stronger regularization, which helps to prevent overfitting, while a larger value weakens the regularization, potentially allowing the model to fit the data more closely; (3) Solver: The optimization algorithm used to minimize the loss function. Common options include ‘newton-cg’, ‘lbfgs’, ‘sag’, and ‘saga’. These algorithms differ in terms of their convergence speed and memory requirements.

*Gradient Boosting (GB)* GB is a powerful ensemble learning technique used in machine learning that leverages the concept of boosting to enhance prediction accuracy. In this method, weak learners, usually decision trees, are sequentially built where each tree tries to correct the errors or residuals of its predecessor. This sequential approach helps in reducing both bias and variance, making GB particularly effective for certain classification and regression tasks [28]. The key hyperparameters for tuning a GB model, along with their respective considered values, are (1) n_estimators: This parameter dictates the number of trees in the ensemble. A higher value might increase the performance but can also prolong the training time and risk overfitting. The considered range for tuning is 50 to 350, incremented by 50; (2) learning_rate: It regulates the influence of each tree on the final prediction. A lower rate necessitates more trees to achieve equivalent accuracy but can potentially provide a more robust model. The considered range for tuning is 0.1 to 1, incremented by 0.1; (3) max_depth: This parameter specifies the maximum depth of individual trees. While deeper trees can capture more intricate patterns in the data, they are also more prone to overfitting. The considered range for tuning is 1 to 25, incremented by 2; 4) max_features: This parameter sets a limit on the number of features assessed for finding the optimal split at each node. Using fewer features can help prevent overfitting, albeit possibly at the cost of performance. The considered values for tuning range from 10 to 100 percent of the number of features, incremented by 10% of the number of features.

*Random Forest (RF)* RF is a widely used ensemble learning technique celebrated for its simplicity and high classification accuracy. In this method, multiple decision trees are constructed during the training phase, and predictions are made by aggregating the outputs of individual trees, usually through a voting mechanism for classification tasks [29]. This strategy not only enhances prediction accuracy but also resists overfitting, which is a common problem in machine learning. Key hyperparameters for tuning a RF model, along with their respective considered values, include (1) n_estimators: Defines the number of trees in the forest. The considerations for tuning this parameter are the same as in GB, focusing on balancing performance improvement with computational efficiency; (2) criterion: Assesses the quality of a split during the construction of the trees. The supported options are Gini impurity and entropy, which help to determine the most informative features for splits; (3) max_depth: Specifies the maximum depth of the trees in the forest. The considerations for tuning this parameter are the same as in GB, typically involving a range that helps prevent overfitting while still capturing important data patterns; (4) max_features: Identifies the quantity of features to evaluate while seeking the optimal division at each node. The considerations for tuning this parameter are the same as in GB, usually involving a range that balances feature diversity with predictive accuracy; (5) min_samples_leaf: Establishes the least count of samples needed to form a leaf node. The considered range for this parameter is 1 to 5, incremented by 1, which helps in controlling the granularity of the trees; (6) min_samples_split: Establishes the minimum number of samples needed to split an internal node. The considered range for this parameter is 1 to 10, incremented by 2, which controls the complexity of the trees.

*eXtreme Gradient Boosting (XGB)* XGB is a highly efficient and flexible GB library that was developed to optimize both computational speed and model performance. It has become a widely popular tool for machine learning competitions and projects, owing to its effectiveness in handling structured data [30]. By building an ensemble of decision trees (often weak predictors) and merging their outputs, XGB can achieve enhanced predictive performance. The core principle behind boosting is to iteratively add new models to the ensemble, with each new model aiming to correct the errors made by the existing models. In XGB, these models are decision trees, and boosting occurs in a series of iterations where each iteration adds a new tree. A distinctive feature of XGB is the incorporation of regularization (both L1 and L2) on the leaf weights, which helps in preventing overfitting by penalizing complex models. The core tuning parameters and their study ranges for XGB are (1) n_estimators: This parameter refers to the number of trees in the ensemble. More trees might enhance performance but can also increase training time and risk of overfitting. The considered range for this parameter is 50 to 350 incremented by 50); (2) learning_rate: Controls the contribution of each tree to the overall prediction. A lower rate might require a larger number of trees to achieve similar results but can potentially produce more robust models. The considered range for this parameter is 0 to1 incremented by 0.1); (3) max_depth: Defines the maximum depth of individual trees. While deeper trees can capture more intricate patterns in the data, they are also more prone to overfitting. The considered range for this parameter is 1 to 25 incremented by 2; (4) colsample_bytree: Represents the fraction of features selected randomly for each tree. Although the default is 1 (all features), using a fraction can introduce randomness, enhancing model robustness and potentially reducing overfitting. The considered range for this parameter is 0.2 to 1 incremented by 0.1; (5) reg_alpha: This is the L1 regularization term on weights, which helps to control the model complexity by penalizing the absolute sizes of the coefficients, thereby aiding in the prevention of overfitting. The considered values for this parameter are 0, 0.5, and 1; (6) reg_lambda: The L2 regularization term on weights emphasizes smaller overall weights, adding an additional layer of control against overfitting. The considered values for this parameter are 0, 0.5, and 1; (7) subsample: Specifies the fraction of the dataset used in each boosting round, introducing randomness, and helping to prevent overfitting. The considered range for this parameter is 0.5 to 0.9 incremented by 0.1).
